# Whole-genome analysis of papillary kidney cancer finds significant noncoding alterations

**DOI:** 10.1371/journal.pgen.1006685

**Published:** 2017-03-30

**Authors:** Shantao Li, Brian M. Shuch, Mark B. Gerstein

**Affiliations:** 1 Program in Computational Biology and Bioinformatics, Yale University, New Haven, Connecticut, United States of America; 2 Department of Urology, Yale School of Medicine, New Haven, Connecticut, United States of America; 3 Department of Molecular Biophysics and Biochemistry, Yale University, New Haven, Connecticut, United States of America; 4 Department of Computer Science, Yale University, New Haven, Connecticut, United States of America; Baylor College of Medicine, UNITED STATES

## Abstract

To date, studies on papillary renal-cell carcinoma (pRCC) have largely focused on coding alterations in traditional drivers, particularly the tyrosine-kinase, Met. However, for a significant fraction of tumors, researchers have been unable to determine a clear molecular etiology. To address this, we perform the first whole-genome analysis of pRCC. Elaborating on previous results on *MET*, we find a germline SNP (rs11762213) in this gene predicting prognosis. Surprisingly, we detect no enrichment for small structural variants disrupting *MET*. Next, we scrutinize noncoding mutations, discovering potentially impactful ones associated with *MET*. Many of these are in an intron connected to a known, oncogenic alternative-splicing event; moreover, we find methylation dysregulation nearby, leading to a cryptic promoter activation. We also notice an elevation of mutations in the long noncoding RNA *NEAT1*, and these mutations are associated with increased expression and unfavorable outcome. Finally, to address the origin of pRCC heterogeneity, we carry out whole-genome analyses of mutational processes. First, we investigate genome-wide mutational patterns, finding they are governed mostly by methylation-associated C-to-T transitions. We also observe significantly more mutations in open chromatin and early-replicating regions in tumors with chromatin-modifier alterations. Finally, we reconstruct cancer-evolutionary trees, which have markedly different topologies and suggested evolutionary trajectories for the different subtypes of pRCC.

## Author summary

Renal cell carcinoma accounts for more than 90% of kidney cancers. Papillary renal cell carcinoma (pRCC) is the second most common subtype of renal cell carcinoma. Previous studies, focusing mostly on the protein-coding regions, have identified several key genomic alterations that are critical to cancer initiation and development. However, researchers cannot find any key mutation in a significant portion of pRCC. Therefore, we carry out the first whole-genome study of pRCC to discover triggering DNA changes explaining these cases. By looking at the entire genome, we find additional potentially impactful alterations both in and out of the protein-coding regions. These newly identified critical mutations from scrutinizing the entire genome help complete our understanding of pRCC genomes. Two alterations we find are associated with prognosis, which could aid clinical decisions. We are also able to unveil mutation patterns, signatures and tumor evolutionary structures, which reflect the mutagenesis processes and help understand how heterogeneity arises. Our study provides valuable additional information to facilitate better tumor subtyping, risk stratification and potentially clinical management.

## Introduction

Renal cell carcinoma (RCC) makes up over 90% of kidney cancers and currently is the most lethal genitourinary malignancy [1]. Papillary RCC (pRCC) accounts for 10%-15% of the total RCC cases [2]. Unfortunately, pRCC has been understudied and there is no current form of effective systemic therapy for this disease. pRCC is further subtyped into two major subtypes: type I and type II based on histopathological features. For many years, the only prominent oncogene in pRCC (specifically, type I) that physicians were able to identify was *MET*, a tyrosine-kinase receptor for hepatic growth factor. An amino acid substitution that leads to constitutive activation and/or overexpression are two mechanisms of dysfunction of *MET* in tumorigenesis. Recently, the Cancer Genome Atlas (TCGA) published its first result on pRCC [3], which greatly improves our understanding of the genomic basis of this disease. Several more genes and pathways were identified to be significantly mutated in pRCC. Nevertheless, a significant portion of pRCC cases remain without any known driver. Therefore, we think it is a good time to explore the noncoding regions of the genome using whole-genome sequencing (WGS). Noncoding regions, often overlooked in cancer studies, have been shown to be actively involved in tumorigenesis [4–6]. Mutations in noncoding regions may cause disruptive changes in both cis- and trans-regulatory elements, affecting gene expression. Understanding noncoding mutations helps fill the missing “dark matter” in cancer research. Meanwhile, it is an open question to the degree which these regions harbor significant driver alterations and here we can address this question in the specific context of pRCC.

Multiple endogenous and environmental mutation processes shape the somatic mutational landscape observed in cancers [7]. Analyses of the genomic alterations associated with these processes give information on cancer development and heterogeneity, shed light on the mutational disparity between cancer subtypes and even indicate potential new treatment strategies [8]. Additionally, genomic features such as replication time and chromatin environment govern mutation rate along the genome, contributing to spatial mutational heterogeneity. Last, while studying mutation patterns, landscape and tumor evolution is possible using data from whole exome sequencing (WXS), whole genome sequencing (WGS) gives richer information on mutation landscape and minimizes the potential confounding effects of exome capture process and driver selection.

In this study, we comprehensively analyzed 35 pRCC cases that were whole genome sequenced along with an extensive set of WXS data on multiple levels. We went from microscopic examination of driver genes to analyses of whole genome sequencing variants, and finally, to an investigation of high-order mutational features. We focused on two aims: exploring potential noncoding drivers and better understanding the heterogeneity of the cancer. First, we focused on *MET*, an oncogene that plays a central role in pRCC, especially in the type I. We found rs11762213, a germline exonic single nucleotide polymorphism (SNP) inside *MET*, predicted cancer-specific survival (CSS) in type II pRCC. We also discovered several potentially impactful noncoding mutations in *MET* promoter and its first two introns. The previous TCGA study identified a *MET* alternate transcript as a driver but without illustrating the etiology [3]. We found that a cryptic promoter from an endogenous long interspersed nuclear element-1 (L1) triggered the alternate isoform expression. Surprisingly, we did not find a significant amount of small structural variations affecting *MET*. Then we went on to cases not as easily explained as those with *MET* alterations. We analyzed about 160,000 noncoding mutations throughout the whole genome and found several potentially high-impact mutations in the noncoding regions. Further zooming out, we discovered pRCC exhibited mutational heterogeneity in both nucleotide context and genome location, indicating underlying interplay of mutational processes. We found methylation was the leading factor influencing mutation landscape. Methylation status drove the inter-sample mutation variation by promoting more C-to-T mutations in the CpG context. APOBEC activity, although infrequently observed, left an unequivocal mutation signature in a pRCC genome but not in ccRCC. Also, we discovered samples with chromatin remodeler alternations accumulated more mutations in open chromatin and early-replicating regions. Lastly, we derived evolutionary trees based on the whole-genome mutation calls for each individual sample. The tree topologies varied, reflecting tumor heterogeneity and correlating with the known tumor subtypes.

## Results

### *1*. An exonic SNP in *MET*, rs11762213, predicts prognosis in type II pRCC

We began with coding variants in the long known driver *MET*. The TCGA study of 161 pRCC patients found 15 samples carrying somatic, nonsynonymous single nucleotide variants (SNVs) in *MET*. By analyzing 117 extra WXS samples (see Methods), we found six more nonsynonymous somatic mutations in six samples (S1 Table). V1110I and M1268T were two recurrent mutations in this extra set. Both of them were observed in the TCGA study as well. Additionally, we found two samples carrying H112Y and Y1248C respectively. H1112Y has been observed in two patients in the original TCGA study cohort and H1118R is a long-known germline mutation associated with hereditary papillary renal carcinoma (HPRC) [9]. Y1248C has been observed in type I pRCC before and the TCGA cohort has a case carrying Y1248H. All mutations occurred in the hypermutated tyrosine kinase catalytic domain of *MET*. Two out of these six samples were identified as type I pRCC while the subtypes of the rest four were unknown.

**Fig 1:**
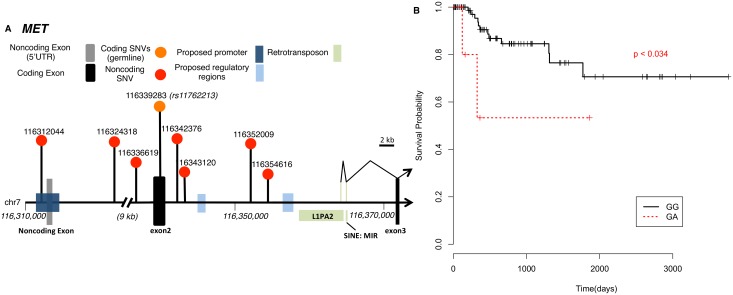
*MET* noncoding alterations and survival analysis of rs11762213 in type II pRCC patients. **(A)** A schematic diagram of noncoding mutations discovered in *MET*. The germline exonic SNP, rs11762213, is also shown. Thin black lines indicate the alternate transcript initiated by a cryptic promoter of an endogenous retrotransponson (L1PA). **(B)** Genotypes of rs11762213 are shown in the legend. Peto & Peto modification of the Gehan-Wilcoxon test.

Although many *MET* somatic mutations are believed to play a central role in pRCC, some germline *MET* mutations have also been associated with the disease. In particular, a germline SNP, rs11762213 (Fig 1A), has been discovered to predict recurrence and survival in a mixed RCC cohort [10]. ccRCC predominated the initial discovery RCC cohort. This conclusion was later validated in a ccRCC cohort but never in pRCC [11]. It was not clear whether this SNP has a prognostic effect in pRCC. Using an extensive WXS set of 277 patients (see Methods; S1 Fig and S1 Table;), we found 14 patients carrying one risk allele of rs11762213 (G/A, minor allele frequency (MAF) = 2.53%). No homozygous A/A was observed. Cancer specific deceases were concentrated in type II pRCC. Among 96 type II pRCC cases, seven patients carried the minor A allele (MAF = 3.65%, Table 1). Cancer-speccific survival was significantly worse in type II patients carrying the risk allele of rs11762213 (p = 0.034, Fig 1B). But we did not find a significant association of this germline SNP with survival in type I patients. We did not observe statistically significant correlation of rs11762213 with *MET* RNA expression in either tumor samples or normal controls (p > 0.1, two-sided rank-sum test). c-Met pY1235 levels in tumor samples, as measured by Reverse phase protein array (RPPA), were not significantly different between carriers of these two genotypes (p > 0.1, two-sided rank-sum test).

**Table 1:** Patient clinical profiles of the type II pRCC cohort in rs11762213 survival analysis. AJCC: American Joint Committee on Cancer; IQR: interquartile range; NA: not available. Percentages may not add up to 100% because of rounding.

Characteristic	G/A (n = 7)	A/A (n = 89)
**Sex, No. (%)**		
Male (%)	4 (57)	25 (28)
Female (%)	3 (43)	64 (72)
**Age, median (IQR), year**	54 (47–61)	65 (57–73)
**Race, No. (%)**		
White	6 (86)	65 (73)
Black	1 (14)	16 (18)
Asian	0	4 (4)
NA	0	4 (4)
**T stage, No. (%)**		
T1	4 (57)	47 (53)
T2	1 (14)	10 (11)
T3	2 (29)	31 (35)
T4	0	1 (1)
**N stage, No. (%)**		
N0	3 (43)	20 (22)
N1	0	15 (17)
N2	1 (14)	2 (2)
NX	3 (43)	52 (58)
**M stage, No. (%)**		
M0	3 (43)	54 (61)
M1	1 (14)	4 (4)
MX/NA	3 (43)	31 (35)
**AJCC stage, No. (%)**		
I	4 (57)	43 (48)
II	0	7 (8)
III	1 (14)	29 (33)
IV	2 (29)	6 (7)
NA	0	4 (4)
**Median follow-up for surviving patients, days (IQR)**	243 (132–354)	579 (219–1247)

### *2*. Epigenetic alterations and mutation hotspots in noncoding regions

The TCGA study identified a *MET* alternate transcript as driver [3]. However, the etiology of this new isoform was unknown. Here we found this alternate transcript resulted from the activation of a cryptic promoter from an endogenous L1 element (Fig 1A), likely due to a local loss of methylation [12]. This event was reported in several other cancers [13, 14]. To test its relationship with methylation, we found the closest probe (cg06985664, ~3kb downstream) on the methylation array showed marginally statistically significantly lower methylation level in samples expressing the alternate transcript (p = 0.055, one-sided rank-sum test). Additionally, this event was associated with methylation cluster 1 (odds ration (OR) = 4.54, p<0.041), indicating genome-wide methylation dysfunction. This association was stronger in type II pRCC and the alternate transcript was tightly associated with the C2b cluster (OR = 17.5, p<0.007).

Despite the fact, *MET* is the most common driver alteration, about 20% presumably *MET*-driven yet *MET* wild-type pRCC samples were still left unexplained [3]. That is, they had a characteristic Met-dysregulated gene expression pattern but no obvious Met-associated alteration. Therefore, we scanned the *MET* noncoding regions. We observed one mutation in *MET* promoter region in a type I pRCC sample (Fig 1A and S2 Table). This sample showed no evidence of nonsynonymous mutation in *MET* gene but had copy number gain of *MET*. Additionally, we observed 6/35 (17.1%) samples carry mutations in the intronic regions between exons 1–3 of *MET* (Fig 1A and S2 Table). As we describe above we could see that these mutations nearby to regions with methylation dysregulation and the activation of a cryptic promoter. However, we were not able to find a direct statistically significant correlation between the alternative splicing event and these intronic mutations.

**Fig 2:**
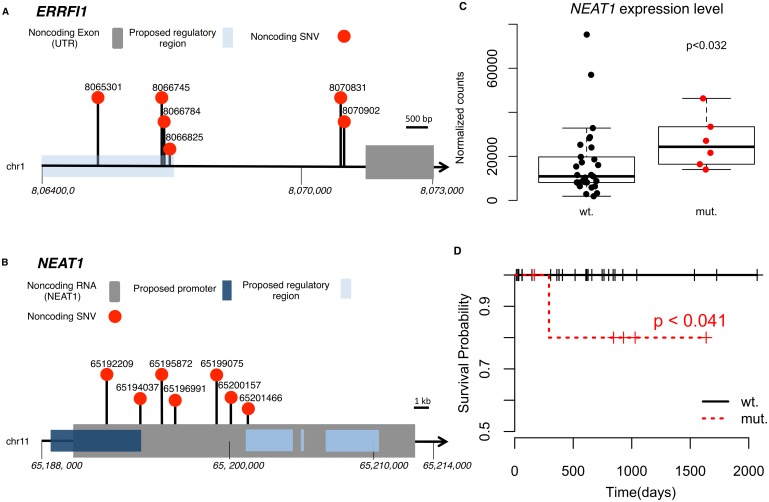
Noncoding alterations in *ERRFI1* and *NEAT1*. **(A)** A schematic diagram of noncoding mutations discovered in *ERRFI1*. **(B)** A schematic diagram of noncoding mutations discovered in *NEAT1*. One tumor carries two mutations on *NEAT1*. **(C)** Tumors with mutations in *NEAT1* show higher *NEAT1* expression. **(D)** Survival analysis shows mutations in *NEAT1* are associated with worse prognosis. Log-rank test.

We further expanded our scope and ran FunSeq [4, 5] to identify potentially high-impact, noncoding variants in pRCC. First, we identified a high-impact mutation hotspot on chromosome 1. 6/35 (17.1%) samples had mutations within this 6.5kb region (Fig 2A and S2 Table). This hotspot located at the 5’-end of *ERRFI1* (ERBB Receptor Feedback Inhibitor 1) and overlapped with the predicted regulatory region. ERRFI1 is a negative regulator of EGFR family members, including EGFR, HER2 and HER3; all have been implicated in cancer. However, due to a limited sample size here, our test power was inevitably low. We did not observe statistically significant changes among mutated samples in mRNA expression level, protein level and phosphorylation level of EGFR, HER2 and HER3.

Another potentially impactful mutation hotspot was in *NEAT1*. We saw mutations inside this nuclear long noncoding RNA in 6/35(17.1%) samples (Fig 2B and S2 Table). Several studies indicated *NEAT1* is associated with various cancers [15, 16]. It promotes cell proliferation in hypoxia [17] and alters the epigenetic landscape, increasing transcription of target genes [18].

Mutations we found all fell into a putative promoter and its flanking region of *NEAT1*. We noticed *NEAT1* mutations were associated with higher *NEAT1* expression (Fig 2C, S2A Fig, p < 0.032, two-sided rank sum test). We also found *NEAT1* mutations were associated with worse prognosis (Fig 2D, p < 0.041, log-rank test). To further investigate the role of *NEAT1* in RCCs, we found *NEAT1* overexpression is significantly associated with shorter overall survival in ccRCC (TCGA cohort, p = 0.0132, S2B Fig). Moreover, *MALAT1*, another noticeable lncRNA in cancer, is tightly co-expressed with *NEAT1* in both pRCC and ccRCC (Spearman’s correlation; 0.79 and 0.87 respectively). *MALAT*1 is located ~50kb downstream of *NEAT1* and might share the same regulatory mechanism with it. The Catalogue of Somatic Mutations in Cancer (COSMIC) [19] annotates *MALAT1* as a cancer consensus gene, associating it with pediatric RCC and lung cancer. *MALAT1* was also reported to be associated with ccRCC [20].

### *3*. Structural variations in pRCC

We performed structural variants (SVs) discovery using WGS reads (see Methods and S3 Table, S2C Fig). This SV discovery approach has higher sensitivity and resolution than array-based copy number variation methods, which were employed in the TCGA analysis. This was a large-scale, big-compute calculation that involves mapping more than 100 billion reads (see Methods). In the end, we found 424 somatic SV events, including 170 deletions, 53 duplications, 105 inversions and 96 translocations (S3 Table). The samples clearly split into two categories based on the number of SV events (ranging from 0 to 88): genome unstable (6 samples, >40 events/per samples) and genome stable (29 samples, <10 events/per sample). The unstable category was significantly associated with type II versus type I pRCC (p<0.015, two-tailed Fisher exact test) and enriched in the C2b cluster (p < 0.002, two-tailed Fisher exact test).

We overlapped SVs with curated cancer genes from COSMIC [19]. Somewhat surprisingly, we did not find SVs affecting *MET* except a single example—one genomically highly unstable sample, TCGA-B9-4116, with deep amplification of *MET*, showed multiple SVs of various classes hitting *MET*. To explain this lack of enrichment for small SVs in *MET*, we postulated trisomy/polysomy 7 is the main mechanism of *MET* structural alteration rather than small-scale duplication. Moreover, besides duplication, we did not expect to find deletion, inversion or translocation disrupting oncogene *MET*. These SVs were likely to cause loss-of-function rather than gain-of-function. Indeed, we did not find any breakpoint splitting *MET*. This was consistent with the putative role of *MET* as an oncogene, rather than a tumor suppressor.

We next looked for other cancer genes affected by somatic SVs. We found two cases with deletions in *SDHB*. The median *SDHB* expression was significantly lower (p<0.0034, one-sided rank sum test), only ~50% compared to cases without alternation (S2D Fig). We validated the deletions affecting *SDHB* with another SV caller, Lumpy-SV. Besides, we confirmed three cases carrying deletions affecting *CDKN2A* called by the TCGA array-based method but not the other two cases. Notably, three confirmed cases had significantly lower *CDKN2A* expression (p<0.0013, one-sided rank sum test) but the unconfirmed two cases did not (S2D Fig). This suggests SV calling from WGS is accurate and predicts *CDKN2A* expression better. Lastly, we observed several high-impact sporadic events, including duplications in *EGFR* and *HIF1A*, and deletions in *DNMT3A* and *STAG2* (S2C Fig).

### *4*. Mutation spectra and mutation processes of pRCC

**Fig 3:**
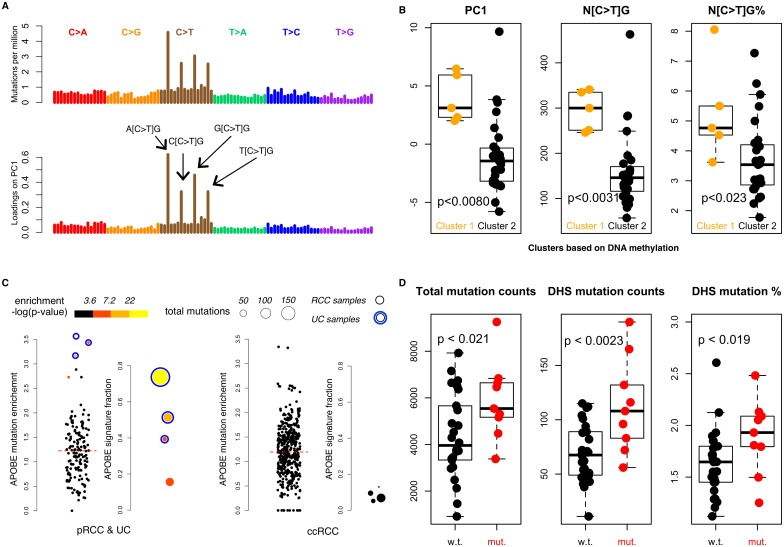
Mutation spectra and mutation processes in pRCC. **(A)** The mutation spectrum of all pRCC WGS samples. Mutations are ordered in alphabetical order of the reference trinucleotides (with the mutated nucleotide in the middle, from A[C>A]A to T[T>G]T) from left to right. Lower panel; we used PCA to maximize inter-sample variation. The loadings on the first principle component are dominated by C>T in CpGs. **(B)** PC1, C>T in CpGs mutation counts and the percentages of such mutations among total mutations are significantly different between two methylation clusters. **(C)** APOBEC mutation signatures are shown for both pRCC (along with three UC samples, blue outer circles) and ccRCC TCGA cohorts. Red dashed line represents the median APOBEC enrichment. **(D)** Mutation counts, mutations counts in open chromatin regions and the percentages of mutations in open chromatin regions are significantly higher in tumors with chromatin remodeling gene alterations compared to the ones without.

To further get an overview of the mutation landscape, we summarized the mutation spectra of 35 whole genome sequenced pRCC samples (Fig 3A). C-to-T in CpGs showed the highest mutation rates, which were roughly three to six-fold higher than mutation rates of other nucleotide contexts.

We used principle components analysis (PCA) to reveal factors that explained the most inter-sample variation. The loadings on the first principle component (which explained 12.5% of the variation) demonstrated C-to-T in CpGs contributed the most to inter-sample variation (Fig 3B). C-to-T in CpGs is highly associated with methylation. It reflects the spontaneous deamination of cytosines in CpGs, which is much more frequent in 5-methyl-cytosines [21]. So we further explored the association between C-to-T in CpGs and tumor methylation status. First, we validated the TCGA identified methylation cluster 1 showed higher methylation level than cluster 2 in all annotated regions (S3 Fig, see Methods), prominently in CpG Islands (Odds ratio of sites being differentially hypermethylated: 1.29, 95%CI: 1.20–1.39, p<0.0001). We confirmed this association by showing samples from methylation cluster 1 had higher PC1 scores as well as higher C-to-T mutation counts and mutation percentages in CpGs (Fig 3C). This trend was further validated using a larger WXS dataset as well. Especially, the most hypermethylated group, CpG island methylation phenotype (CIMP), showed the greatest C-to-T rate in CpGs (S3C Fig). Therefore, methylation status was the most prominent factor shaping the mutation spectra across patients.

Furthermore, we explored the functional impacts of the excessive mutations driven by methylation. C-to-T mutations in CpGs we observed in pRCCs were more likely to be in the coding region (OR = 1.54, 95%CI: 1.27–1.85, p<0.0001) and nonsynonymous (OR = 1.47, 95%CI: 1.17–1.84, p<0.001), which indicated they tended to be high-impact mutations. However, C-to-T mutations in CpGs did not show functional bias between the two methylation clusters in noncoding regions (based on FunSeq score distribution).

Recently, 30 somatic mutation signatures were identified; many have putative etiology, revealing the underlying mutational processes and helping understand tumor development [7]. We used a LASSO-based approach (see Methods) to decompose the observed mutations into a linear combination of these canonical mutation signatures in both WGS and WXS samples (S4 Fig). The leading signature was "signature 5" (from reference 7). Interestingly, we found one type II pRCC case out of 155 somatic WXS sequenced samples exhibited APOBEC-associated mutation signatures 2 and 13. APOBEC mutation pattern enrichment analysis (see Method) further confirmed the presence of APOBEC activity (Fig 3D, S4 Table). This sample was statistically enriched of APOBEC-induced mutations (adjusted p-value < 0.0003).

Prominent APOBEC activities were also incidentally detected in three upper track urothelial cancer (UC) samples sequenced and processed in the same pipeline with pRCC samples. UC often carries APOBEC associated mutation signatures and our result is consistent with the TCGA bladder urothelial cancer study [22].

The APOBEC associated signature carrying pRCC case was centrally reviewed by six pathologists in the original study and confirmed to be type II pRCC [3]. Thus, this tumor is likely a special case of type II with genomic alterations sharing some similarities with UC. It had non-silent mutations in *ARID1A* and *MLL2* and a synonymous mutation in *RXRA*, all are identified as significantly mutated genes in UC but not in pRCC. Potential type II pRCC driver events, for example, low expression of *CDKN2A* and nonsynonymous alternations in significantly mutated genes of pRCC, were absent in this sample. Noticeably, the four samples with APOBEC activities showed significantly higher *APOBEC3A* and *APOBEC3B* mRNA expression level (p < 0.0022 and p < 0.0039 respectively, one-sided rank sum test, S5 Fig). This is in concordance with previous studies of APOBEC mutagenesis in various types of cancer [23].

Consistent with previous studies [12], we failed to detect statistically significant APOBEC activities in an extensive WXS dataset of 418 clear cell RCC (ccRCC) samples, even after subsampling to avoid p-value adjustment eroding the power. Very low levels of APOBEC signatures (<15%) were found in less than 1%(4/418) samples. With a much larger sample size, this result was unlikely to be confounded by detecting power.

### *5*. Defects in chromatin remodeling affect mutation landscape

Chromatin remodeling genes are frequently mutated in pRCC and many other cancers, including ccRCC [3, 24, 25]. Defects in chromatin remodeling cause dysregulation of the chromatin environment. Open chromatin regions usually show a lower mutation rate, presumably due to more effective DNA repair [26]. Thus, chromatin remodeler alterations could possibly alter the mutation landscape, specifically increasing mutation rate in previously open chromatin regions. To test this, we tallied the number of mutations inside DNase I hypersensitive sites (DHS) inferred from 11 normal fetal kidney cortex samples (The NIH Roadmap Epigenomics Mapping Consortium) [27], which represent normal tissues under physiological conditions. 9/35 samples with disruptive mutations in ten chromatin remodeling genes, cancer-associated genes showed higher genome-wide mutation counts (p < 0.021, one-sided rank-sum test;), partially driven by higher mutation counts in the DHS regions (p < 0.0023, one-sided rank-sum test). The median number of mutations in DHS regions considerably increased by 60% (67.5 versus 108) in samples carrying chromatin remodeling defects. The effect was still significant after normalizing against the total mutation counts (p < 0.019, one-sided rank-sum test, Fig 3E), indicating a significant shift in mutation landscape.

Replication time is known to correlate greatly with mutation rate. Early replicating regions have lower mutation rate compared to late replicating ones. Researchers reason replication errors are more likely to be corrected by DNA repair system in early replicating regions. With defects in chromatin remodeling genes, we observed this trend became less pronounced (p<0.031, one-sided rank-sum test, S6 Fig). This is presumably because dysregulation of the chromatin environment hinders replication error repair by changing the accessibility of newly synthesized DNA chains.

### *6*. Evolutionary trees reveal the heterogeneity of tumor evolutionary profile

**Fig 4:**
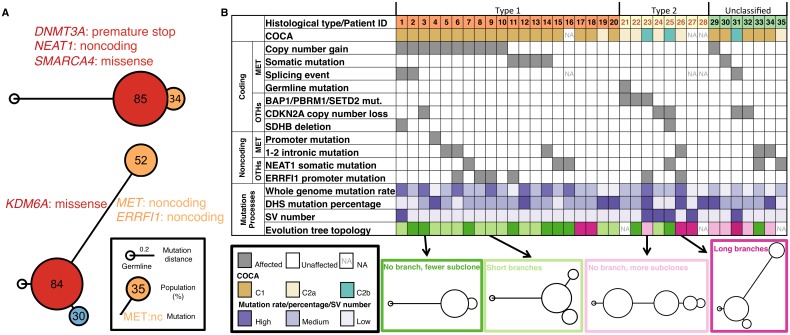
Evolutionary trees and genomic alteration landscape of 35 pRCC WGS samples. **(A)** Two individual evolutions trees. Mutations in cancer-related gene are shown in colors corresponding to where they first appear. **(B)** Summary table of alterations in 35 pRCC WGS. Index: patient index, see S2 table.

With the richness of SNVs in WGS samples, we can further tackle the mutational process heterogeneity of pRCC by constructing evolutionary trees for the 35 tumors (S7 Fig). These trees were derived from the whole-genome mutation calls and were produced individually for each tumor, with their topology suggesting a temporal ordering to the mutations. We could classify the trees into four groups based on their topology (Fig 4):

**Group 1,** no branch, fewer subclones (10, 32.3%),
**Group 2,** short branches (12, 37.5%),
**Group 3,** no branch, more subclones (5, 15.6%) and
**Group 4,** long branches (5, 15.6%).

In addition, three trees were excluded from the analysis since they had a largest population faction <0.5, which was likely due to low mutation number, high sequence error and/or particularly high copy number variation.

Both topology groups 3 and 4 showed significant clonal evolution, with more distal subclones, and greater heterogeneity, indicated by substantial mutational divergence between populations. These groups were significantly depleted in type I pRCC (p < 0.0034, two-tailed fisher exact test). In contrast, the short branch group (#2) was significantly enriched in type I pRCC (p<0.011, two-tailed fisher exact test, Fig 4B). This suggested type I tumors were more homogenous and showed less complex evolutionary features compared to type II and unclassified samples.

## Discussion

Our study is the first one that comprehensively looked into the noncoding regions of pRCC. Doing so allowed us to tackle an open question in the field of cancer genomics, whether whole genome sequencing adds additional value over whole exome sequencing. We comprehensively analyzed both WGS and an extensive set of WXS of pRCC, scrutinizing local high-impact events as well as giving an overall view of the mutation landscape and evolution. Our work further completed the genomic alteration landscape of pRCC (Fig 4B). Beyond traditionally driver events, we suggested several novel noncoding alterations potentially drive tumorigenesis. We also provided valuable insights to tumor heterogeneity though investigating the mutational patterns, landscape, and evolutionary profiles.

First, we elaborated on previous results of the long known driver *MET*. In an extended 117 WXS dataset, we found six additional nonsynonymous somatic mutations in the hyper-mutated tyrosine kinase catalytic domain. These somatic mutations were highly recurrent, concentrated on a few critical amino acids. This was in line with *MET* being an oncogene and supported its central driver status in pRCC. Then we found an exonic SNP in *MET*, rs11762213, to be a prognostic germline variance in type II pRCC. Previously, rs11762213 was found to predict outcome in a mixed RCC samples, predominated by ccRCC [10]. Later, the result was confirmed in a large TCGA ccRCC cohort [11]. However, it was never clear whether rs11762213 only predicts the outcome in ccRCC or other histological types as well. In this study, we concluded that the minor alternative allele of rs11762213 also forecasts unfavorable outcome in type II pRCC patients. The mechanism of this exonic germline SNP remains unsettled. A previous study proposed it disrupts a putative enhancer of *MET* [11]. However, researchers could not find significant association between the SNP and *MET* expression in either tumor or normal tissues. We noticed there is no other gene within 100 kb in both directions of this SNP. Given the significant role of *MET* in pRCC, we think rs11762213 is affecting survival through *MET*, although the mechanism unknown.

Similar to ccRCC, type II pRCC is not primarily driven by *MET*. Not as significantly mutated in ccRCC and type II pRCC, *MET* nonetheless seems to play a role in cancer development. Our finding on rs11762213 is potentially meaningful in the clinical management of patients with the more aggressive type II pRCC. rs11762213 genotyping could become a reliable, low-cost risk stratification tool for these patients. Also, rs11762213 might become a biomarker for predicting response to Met inhibitors pending further studies.

Interestingly, rs11762213 is prevalent mostly in European and American populations but not in African populations and rare in Asian populations. However, the MAF of rs11762213 among African American patients in our cohort is 2.73%, higher than MAFs in general for African populations observed in 1000 Genome phase 3 dataset (0.2%, with 0% in Americans with African ancestry, ASW) [28] and the ExAC dataset (1.1%, excluding TCGA cohorts) [29]. This implies a possible effect of rs11762213 on pRCC incidence among African Americans that is worth further investigation.

In *MET* noncoding regions, we first found a cryptic promoter from a retrotransposon in the second intron initiates the alternate transcript, which was classified as a driver by the TCGA study (3). Methylation is a major source of silencing retrotransposon activities in the human genome [12–14]. Indeed, we observed evidence for a local loss of methylation and global methylation dysregulation in samples expressing the alternate transcript. Our finding indicates methylation change might directly drive pRCC growth through *MET*.

We also discovered mutations associated with the *MET* promoter and first two introns, where the alternate transcript starts. Although the implication is unknown, our analysis suggests there is a mutation hotspot in *MET* that calls for further research.

Expanding our scope from coding to non-coding and using FunSeq to group SNVs by functional elements, we found several potentially significant noncoding mutation hotspots relevant to tumorigenesis throughout the entire genome. A mutation hotspot was found downstream of *ERRFI1*, an important regulator of the EGFR pathway, which may serve as a potential tumor suppressor. EGFR inhibitors have been used in papillary kidney cancer with an 11% response rate observed [30]. These mutations potentially disrupt regulatory elements of *ERRFI1* and thus play a role in tumorigenesis. However, likely limited by a small sample size, we were not able to detect statistically significant functional changes in ERRFI1 and related pathways. Another noncoding hotpot was in *NEAT1*, a long noncoding RNA that has been speculated to involve in cancer. Patients carrying mutations in *NEAT1* had significantly higher *NEAT1* expression and worse prognosis. High expression of *NEAT1* predicted significantly worse survival in ccRCC as well. *NEAT1* has been shown to be hypermutated in other cancers and some studies also linked high *NEAT1* association with unfavorable prognosis [31, 32]. Lastly, a downstream lncRNA, *MALAT1*, showed tight co-expression pattern with *NEAT1* in both pRCC and ccRCC. *MALAT1* is on COSMIC consensus cancer gene list and annotated as related with pediatric RCC [19]. It was also reported to be associated with ccRCC [20].

Next, with more than 100 billion carefully remapped reads from WGS, we generated a high-confident SV dataset for 35 pRCC samples. Our method has great accuracy. In fact, we confirmed the well-known deletion of *CDKN2A* and found that we predicted its down-regulate expression better than the copy number variation analysis in TCGA study [3].

In terms of overall numbers of SVs, we found the pRCCs clearly split into two categories: the stable category had less than 10 events per sample while the unstable category had all above 40. Moreover, the unstable category was tightly associated with the C2b cluster, which has inferior outcomes [3]. Our SV study also discovered recurrent cases of *SDHB* deletion and expression data supported our finding. SDHB is a subunit of succinate dehydrogenase. Previous studies indicated the loss of SDHB being a driver event by disturbing tumor metabolic environment [33, 34] Besides *SDHB*, we also found some other sporadic events involving known tumor drivers.

Somewhat counter-intuitively, we found the absence of *MET* alterations that involve small deletion or breakage of the *MET* gene except in one highly unstable sample. Large-scale duplications involving *MET*, however, have been found (e.g. trisomy 7). This finding can be rationalized by realizing that the oncogenic activity of MET is encouraged by amplification but not by deletion or disruption. Moreover, we postulated that polysomy 7 might be the major mechanism of *MET* gain and lack of smaller SVs and breakpoints disrupting *MET* further supports its oncogene role.

WGS provides many times more SNVs compared to WXS. Thus it gives us an opportunity to look into the high-level landscape of mutations in pRCC. Several recent landmark pan-cancer studies lead to the wide recognition of significance and great research interests in cancer mutational processes [7, 8, 26, 35, 36]. DNA mutation is one of the driving forces of cancer development, and understanding the underlying processes and affecting factors that generate the mutations is vital in cancer studies. In particular, we focused on revealing the underlying sources that fuel tumor heterogeneity, a key feature of pRCC.

We identified mutation rate dispersion of C-to-T transitions in CpGs motifs contributed the most to the inter-sample mutation spectra variation. We further pinned down the cause of dispersion by showing the hypermethylated cluster, identified in the previous TCGA study [3], had a higher C-to-T rate in CpGs. Although increased C-to-T in CpGs is likely the result of hypermethylation, we cannot rule out the possibility the change of mutation landscape plays a role in cancer development. For example, C-to-T in methylated CpGs causes loss of methylation, which could have effects on local chromatin environment, trans-elements recruitment and gene expression regulation. In our study, we observed C-to-Ts in CpGs were enriched in coding regions, which suggested they might have a higher functional impact in the cancer genome.

Significant APOBEC activities and consequential mutation signatures were observed in one type II pRCC case. APOBEC activities were known to be prevalent in UCs [22, 23]. We also successfully detected prominent APOBEC signatures in all three UC samples processed in the same pipeline as pRCCs. Intriguingly, despite being considered to have the same cellular origin with pRCC, we were not able to detect meaningful APOBEC activities in ccRCC. This was in agreement with previous studies [12]. APOBEC mutation signature was also found in a small percentage of chromophobe renal cell carcinoma [37], although they are believed to have a different cellular origin. APOBEC activities have been linked with genetic predisposition and viral infection [38]. Given a statistically robust signal in our conservative algorithm, it is plausible that a small fraction of type II pRCCs might share some etiologically and gnomically similarities with UC. Standard treatment for UC differs significantly from the one for pRCC. Pending further research, this finding might suggest actionable clinical implications.

The chromatin remodeling pathway is highly mutated in pRCC [3, 24, 25]. Several chromatin remodelers have been identified as cancer drivers in pRCC. We investigated the relationship between samples with mutated chromatin remodelers and those without such mutations in terms of mutation landscape. We demonstrated pRCCs with defects in chromatin remodeling genes showed higher mutation rate in general, driven by an even stronger mutation rate increase in putative open chromatin regions in normal kidney tissues. This is likely because chromatin remodeling defects disrupt normal open chromatin environment and impede DNA repairing in these regions.

It is known that replication time strongly governs local mutation rate. Early replication regions have fewer mutations. But the difference dissipates when DNA mismatch repair becomes defective [21]. In our study, we found this correlation weakened in samples with mutated chromatin remodeling genes, presumably caused by failure of replication error repair in an abnormal chromatin environment. Through defects in chromatin remodeling genes, a tumor alters its mutation rate and landscape, which might provide it advantage in cancer evolution. Yet, high mutation burden in functional important open chromatin regions also raises the chance that tumor antigens activate the host immune system. Researchers found tumors with DNA mismatch repair deficiency responded better to PD-1 blockage [39]. These tumors also accumulate more mutations in early replicating regions [26]. Thus chromatin remodeler alterations might as well correlate with higher response rate of immunotherapy, which is worth further studies.

Finally, we constructed individual evolutionary trees for all 35 samples. This is the first study inferring tumor evolutionary trees using a large number of SNVs from WGS in pRCC. Benefited from a large number of SNVs, the tree construction became more statistically robust and revealed more details. In general, evolutionary trees gave us the opportunity to observe how pRCC heterogeneity developed over time. They revealed the history of the tumor and how mutations accumulated. We discovered the trees exhibited four major types of topologies, reflecting different levels of heterogeneity. Type II pRCCs showed distinct evolutionary topologies from type I, perhaps indicating an association with greater heterogeneity and different evolving trajectories.

In this first whole genome study of pRCC, we found several novel noncoding alterations that might drive tumor development and we explored the mutational landscape and evolutionary trees to better understand tumor heterogeneity. However, due to a limited sample size, some of our statistical tests were underpowered. As the cost of sequencing keeps dropping and technology for data management and processing continues advancing, we expect to have more whole genome sequenced tumors in the near future [40]. With a larger cohort, we hope to gain enough power to test the hypotheses we formed as well as further explore the noncoding regions of pRCC.

## Materials and methods

### Data acquisition

pRCC and ccRCC WXS and pRCC WGS variants calls were downloaded from the TCGA Data Portal (https://gdc-portal.nci.nih.gov/legacy-archive/search/f) and TCGA Jamboree (https://tcga-data-secure.nci.nih.gov/tcgafiles/tcgajamboree) respectively. pRCC RNAseq, RPPA and methylation data (under project ID: TCGA-KIRP) were downloaded from TCGA Data Portal as well. Wavelet-smoothed repli-seq data was obtained as a part of ENCODE project [41–43] and downloaded from UCSC Genome Browser (Also accessible under GSE34399 in the Gene Expression Omnibus). DHS data (fetal, kidney cortex) were obtained from Roadmap Epigenomics Project and are accessible from http://www.genboree.org/EdaccData/Current-Release/sample-experiment/Fetal_Renal_Cortex/Chromatin_Accessibility/.

### Testing rs11762213 on prognosis and exploring somatic mutations in *MET*

We downloaded pRCC clinical outcomes from TCGA Data Portal (https://tcga-data.nci.nih.gov/tcga/tcgaDownload.jsp). pRCC samples that failed the histopathological review were excluded [3]. In total, we included 277 patients in our analyses (S1 Fig, S1 Table). For germline calls, the majority of samples, 163 out of 277, were supported by germline SNV callings from two centers (BCM and BI). 100% genotype concordance rate was observed. Also, 162 curated rs11762213 genotypes were in agreement with automated call sets. All calls have alternative allelic fraction of 0.42 to 0.68, supporting heterozygous genotype [11]. Calls from BI all have genotype quality scores >125 and all calls in BCM pass the caller filter. With proved high confidence in the accuracy of genotyping rs11762213 in the germline, we recruited additional 114 samples from single-center (BCM), automated calls to form an extensive patient set (S1 Fig). For somatic SNVs in *MET*, after excluding cases that were recruited in the TCGA study, we formed an additional set encompassing 117 patients. Five callings were supported by two centers. The rest were supported by single-center (BCM) automated calls.

Cancer-specific survival was defined using the same criteria as described in a ccRCC study [9]. Deaths were considered as cancer-specific if the “Personal Neoplasm Cancer Status” is “With Tumor”. If “Tumor Status” is not available, then the deceased patients were classified as cancer-specific death if they had metastasis (M1) or lymph node involvement (≥ N1) or died within two years of diagnosis. An R package, “survival”, was used for the survival analysis.

### SV calling procedure

We remapped all reads using bwa 0.7.12, which supports split read mapping [44]. Then we used DELLY [45] with default parameters for somatic SV calling. To avoid sample contamination or germline SVs, we filtered our call set against the entire TCGA pRCC WGS dataset, regardless of sample match. We discharged all callings that were marked “LowQual” (PE/SR support below 3 or mapping quality below 20). Last, to further eliminate germline contamination, we filtered out SVs that show at least 0.8 reciprocally overlapping with 1000 Genome Phase 3 SV call set (only 1/425 filtered out).

For Lumpy-SV [46], we ran it with default parameters. We also filtered the results using the 1000 Genome Phase 3 call set and required the SV have both paired-end and split reads supports.

### Mutation spectra study

WGS Mutations were extracted with flanking 5’ and 3’ nucleotide context. The raw mutation counts were normalized by trinucleotide frequencies in the whole mappable genome.

To identify signatures in the mutation spectra, we used a robust, objective LASSO-based method. First, 30 known signatures were downloaded from COSMIC (http://cancer.sanger.ac.uk/cosmic/signatures). Then we solved a positive, zero-intercept linear regression problem with L1 regularizer to obtain signatures and corresponding weights for each genome. Specifically, we solved the problem:

$$\underset{W}{\text{min}}(∥SW-M{∥}_{2}+\;\lambda ∥W∥)$$

Where *M* is the mutation matrix, containing the mutations of each sample in 96 nucleotide contexts. *S* is the 96×30 signature matrix, representing the mutation probability in 96 nucleotide contexts of the 30 signatures. *W* is the weighting matrix, representing the contribution of 30 signatures to each sample.

The penalty parameter lambda (*λ*) was determined empirically using 10-fold cross-validation individually for every sample.*λ* was chosen to maximize sparsity and constrained to keep mean-square error (MSE) within one standard error of its minimum. Last, we discharged signatures that composite less than 5% of the total detectable signatures.

### Methylation association analysis

In total, we collected HumanMethylation450 BeadChip array data for 139 samples that are either methylation cluster 1 or 2. We used an R package “IMA” to facilitate analysis [47]. After discharging sites with missing values or on sex chromosomes, we obtained beta-values on 366,158 CpG sites in total. Then we tested beta-values of each site by Wilcoxon rank sum test between two methylation clusters. After adjusting p-value using Benjamini-Hochberg procedure, we called 9,324(2.55%) hypermethylation sites. These sites had an adjusted p-value of less than 0.05 and mean beta-values in methylation cluster 1 were 0.2 or higher than in methylation cluster 2.

### APOBEC enrichment analysis

We used the method described by Roberts et al. [23]. For every C>{T,G} and G>{A,C} mutation we obtained 20bp sequence both upstream and downstream. Then enrichment fold was defined as:

$$Enrichment\;Fold=\;\frac{Mutation_{\text{TCW}/\text{WGA}}\;\times \;Context_{C/G}}{Mutation_{C/G}\times Context_{TCW/WGA}}$$

Here TCW/WGA stands for T[C>{T,G}]W and W[G>{A,C}]A. W stands for A or T. p-value for enrichment were calculated using one-sided Fisher-exact test. To adjust for multiple hypothesis testing, p-values were corrected using Benjamini-Hochberg procedure.

WXS data for APOBEC enrichment and signature analysis was obtained from a processed somatic call set: hgsc.bcm.edu_KIRP.IlluminaGA_DNASeq.1.protected.maf. This dataset includes 155 pRCC samples and three UC samples. We used hgsc.bcm.edu_KIRC.Mixed_DNASeq.1.protected.maf for ccRCC analyses.

### Chromatin remodeling genes and replication time association

We identified chromatin remodeling genes based on its significance in pRCC and function. Our gene list was the intersection of genes in the original TCGA pRCC study [3] molecular feature table with the chromatin remodeling and SNI/SWF pathway gene lists. Our gene set included ten genes: *SETD2*, *KDM6A*, *PBRM1*, *SMARCB1*, *ARID1A*, *ARID2*, *MLL2 (KMT2D)*, *MLL3(KMT2C)*, *MLL4(KMT2B)*, *EP300*. We defined chromatin remodeling defect as nonsynonymous mutations in these genes. For missense mutations, we additionally filtered out mutations with Polyphen score [48] less than 0.9 (benign). We noticed *BAP1* is not in the gene list. However, adding *BAP1* into the list did not change the significance of our key tests (p<0.0115 for mutation counts in DHS and p<0.020 for mutation percentage in DHS).

For replication time, in order to avoid cell type redundancy, we only kept GM12878 as the representative of all lymphoblastoid cell lines. Eleven cell types were included in our analysis: BG02ES, BJ, GM12878, HeLaS3, HEPG2, HUVEC, IMR90, K562, MCF7, NHEK, SK-NSH. Wave smoothed replication time signal was averaged in a±10kb region from every mutation. To avoid potential selection effects, we removed mutations in exome and flanking 2bp. Regions overlapping with reference genome gaps and DAC blacklist (https://genome.ucsc.edu/) were removed as well. Last, we picked the median number from 11 cell types at each mutation position.

To test the significance of replication time of noncoding mutations between two groups, we defined the ones have replication time stand above 90 percentile in all pooled mutations as “mutations in early replicating regions”. Then we calculated the percentage of “mutations in early replicating regions” of total mutations for each sample and compared between the two groups using one-sided rank-sum test.

### Evolutionary tree inference

We used PhyloWGS [49] to infer the evolutionary trees for each individual tumor. To mitigate the effects of copy number change, we removed all the SNVs inside the copy number change regions as defined by the assay-based method in the original TCGA study [3]. To be prudent, we filtered SNPs in any region with an absolute log2 tumor to normal copy number ratio larger than 0.3. Additionally, we removed all SNVs with allele frequency higher than 0.6 as they were likely affected by copy number loss.

## Supporting information

S1 Fig Extended WXS dataset acquisition. An extended WXS dataset of 277 patients were obtained from call sets from two different centers. 100% genotyping concordance was observed for germline rs11762213 in cases of multiple center calling results.
(TIF)

S2 Fig *NEAT1* correlated with survival & SV discovery and analysis. **A.** Schematics for *NEAT1* survival study. 35 pRCC patients with *NEAT1* mutation have significantly higher *NEAT1* expression and worse prognosis (see Fig 2C and 2D). **B.** We defined expression >2 standard deviations as high expression and found 5% of ccRCC patients had high *NEAT1* expression level [1,2]. Those patients had significantly worse survival (p = 0.0132, log-rank test, median months of overall survival (OS): 36 versus 77). However, without assessing the mutation status, *NEAT1* expression was not directly significantly correlated with survival in an extended TCGA pRCC cohort. **C.** Schematics for read remapping, SVs calling and prioritization (by overlapping with known cancer genes). The list shows cancer-relevant events we identified. **D.** The expression levels of *SDHB* and *CKDN2A* were significantly lower in samples with deletions. One-sided rank sum test. For *CKDN2A*, TCGA called two other deletions events (blue dots) from array based methods that we could not confirm using our SV pipeline.
(TIF)

S3 Fig Methylation analyses of pRCC. **A.** Volcano plot of all CpG probe sites between methylation cluster 1 and 2. Differences in mean beta values are shown on x-axis and log transformed p-values (rank sum test) are shown on y-axis. Red dashed line represents 0.05 significance level. **B.** Volcano plot of CpG probe sites between methylation cluster 1 and 2 after grouped by functional regions. Differences in mean beta values are shown on x-axis and log transformed p-values (rank sum test) are shown on y-axis. Red dashed line represents 0.05 significance level. Annotation details please refer to the R “IMA” package [3]. **C.** Comparison of C>T in CpGs mutation counts (per millions) and fractions in pRCC WXS set among three different methylation clusters. CIMP: CpG island methylation phenotype. Cluster 1 versus Cluster 2, p < 0.013; CIMP versus Cluster 2: p < 0.02 (rank sum test).
(TIF)

S4 Fig Signatures detected in WXS samples. Upper panel: pie chart of signatures contribution percentages by pooling all samples. Signatures contribute less than 5% were not shown. Lower panel: bar plot shows signature distribution in each individual sample. The results grossly agreed with previous results [4] with minor disparity in signature 3. A few samples with no detectable signature are not shown, mostly because they have too few mutations.
(TIF)

S5 Fig Samples with APOBEC signatures show higher *APOBEC* expression level. The expression levels of *APOBEC3A* and *APOBEC3B* are significantly higher in samples carry APOBEC signatures (red) than the ones do not (blue). p < 0.0022 and p < 0.0039 respectively, one-sided rank sum test.
(PDF)

S6 Fig Mutation rate rises in early replicating regions in chromatin remodeling defected samples. Compared to “wild type” tumors (black), samples with chromatin remodeling gene mutations (orange) had higher percentage of mutations in the early replicating regions. One-sided rank sum test.
(TIF)

S7 Fig Individual evolutionary trees. Frame colors indicates four different topology types (See Fig 4). Mutations in cancer-related gene are shown in colors corresponding to where they first appear. Three trees without frame are the ones with a largest population fraction <0.5, indicating unreliable inference of tree structures (due to low mutation counts, sequence error and/or particularly high copy number variation etc.). They were excluded from downstream analysis.
(TIF)

S1 Table Extra somatic mutations in *MET* found among 111 additional pRCC cases and rs11672213 genotype and cancer-specific survival (CSS) of all 277 patients, including 96 type 2 patients. (XLSX)

S2 Table Molecular summary (non-coding region mutations, mutation fraction in DHS regions etc.) of 35 WGS patients. (XLSX)

S3 Table Structural variants found by DELLY using WGS data. (XLSX)

S4 Table APOBEC mutation signatures and pattern enrichment analysis using WXS data. (XLSX)

S1 Reference References for supplemental files. (DOCX)
