# Supplementary figures and images for: Whole-genome analysis of papillary kidney cancer finds significant noncoding alterations
Source: PLoS Genet. 2017 Mar 30;13(3):e1006685. doi: 10.1371/journal.pgen.1006685 (PMC5391127; doi:10.1371/journal.pgen.1006685)

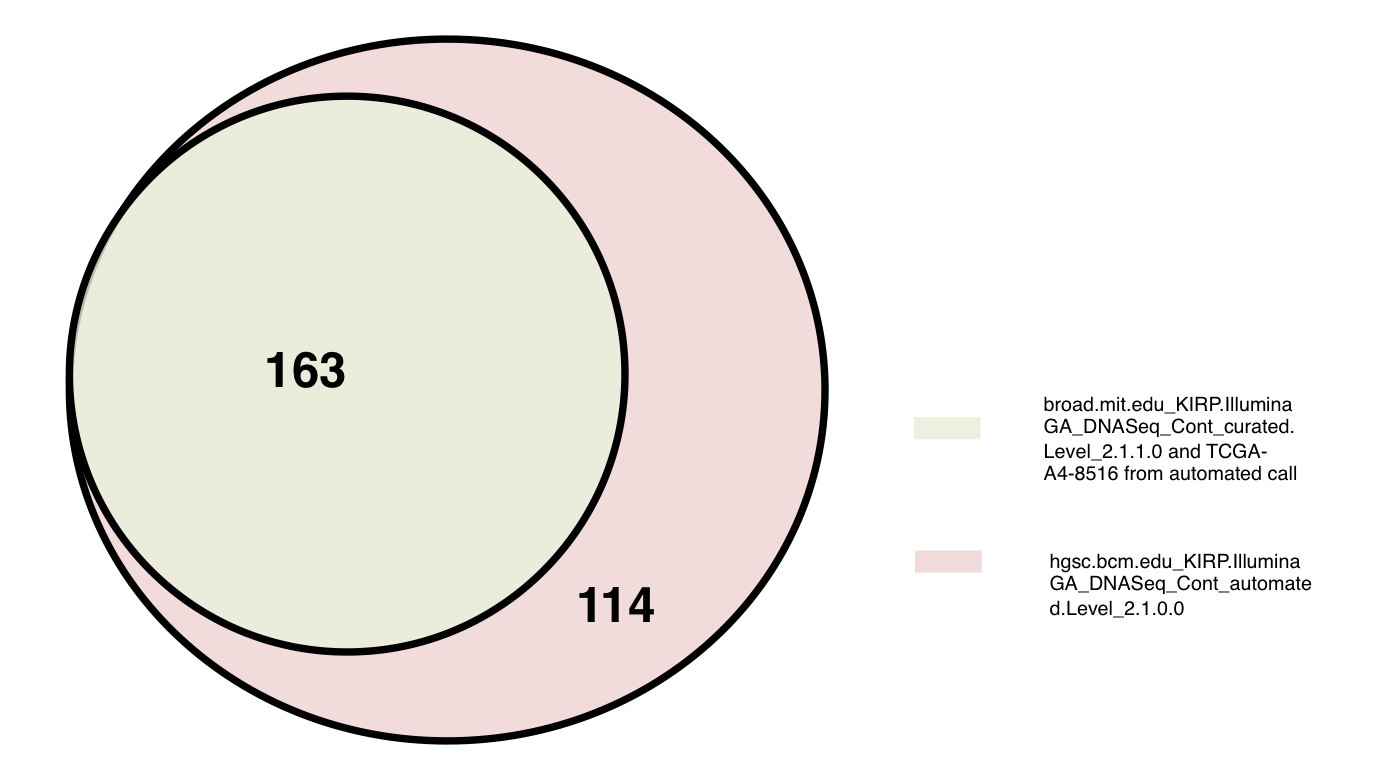

Supplement: S1 Fig — An extended WXS dataset of 277 patients were obtained from call sets from two different centers. 100% genotyping concordance was observed for germline rs11762213 in cases of multiple center calling results. (TIF) [file pgen.1006685.s001.tif]

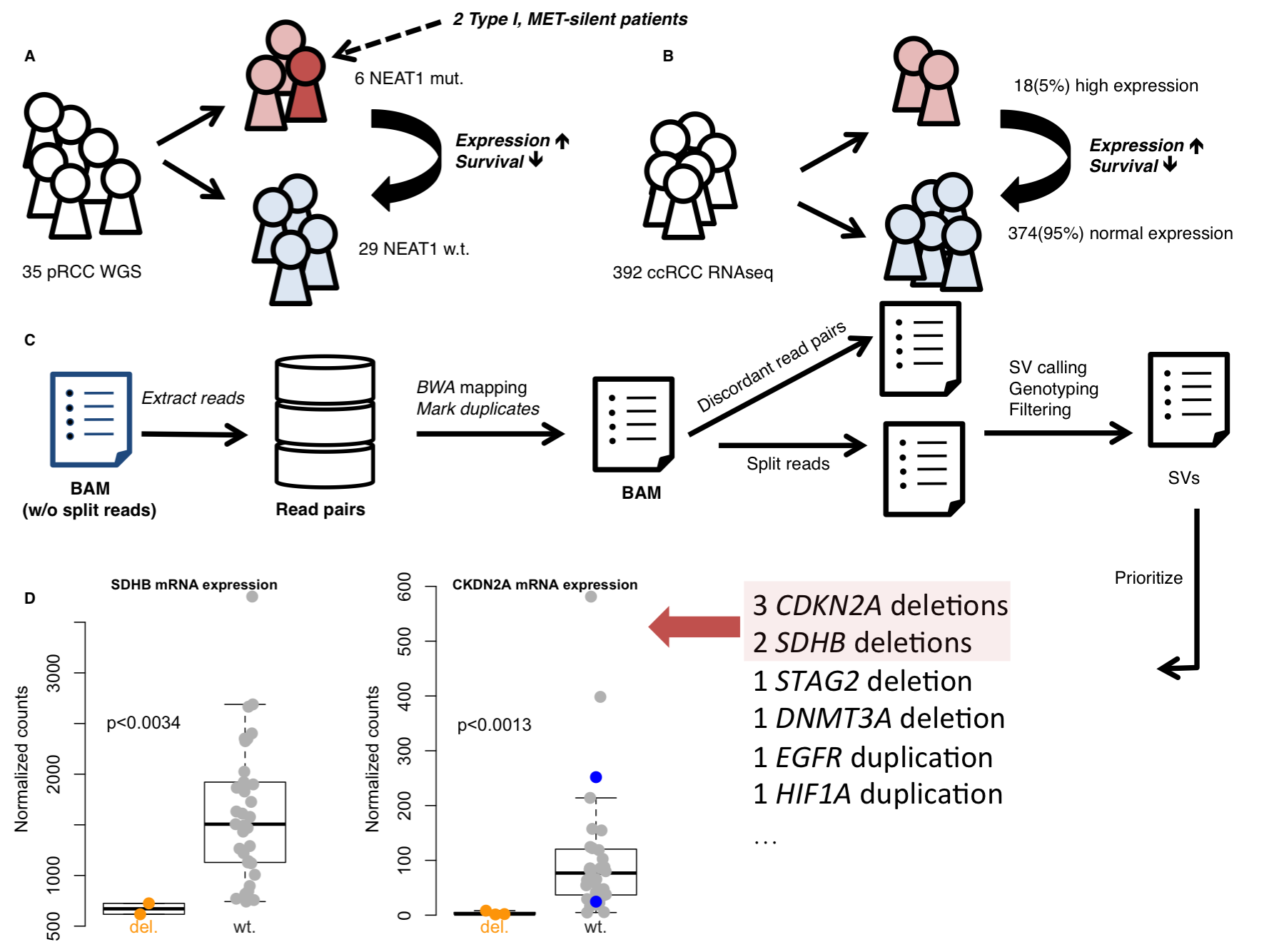

Supplement: S2 Fig — A. Schematics for NEAT1 survival study. 35 pRCC patients with NEAT1 mutation have significantly higher NEAT1 expression and worse prognosis (see Fig 2C and 2D). B. We defined expression >2 standard deviations as high expression and found 5% of ccRCC patients had high NEAT1 expression level [1,2]. Those patients had significantly worse survival (p = 0.0132, log-rank test, median months of overall survival (OS): 36 versus 77). However, without assessing the mutation status, NEAT1 expression was not directly significantly correlated with survival in an extended TCGA pRCC cohort. C. Schematics for read remapping, SVs calling and prioritization (by overlapping with known cancer genes). The list shows cancer-relevant events we identified. D. The expression levels of SDHB and CKDN2A were significantly lower in samples with deletions. One-sided rank sum test. For CKDN2A, TCGA called two other deletions events (blue dots) from array based methods that we could not confirm using our SV pipeline. (TIF) [file pgen.1006685.s002.tif]

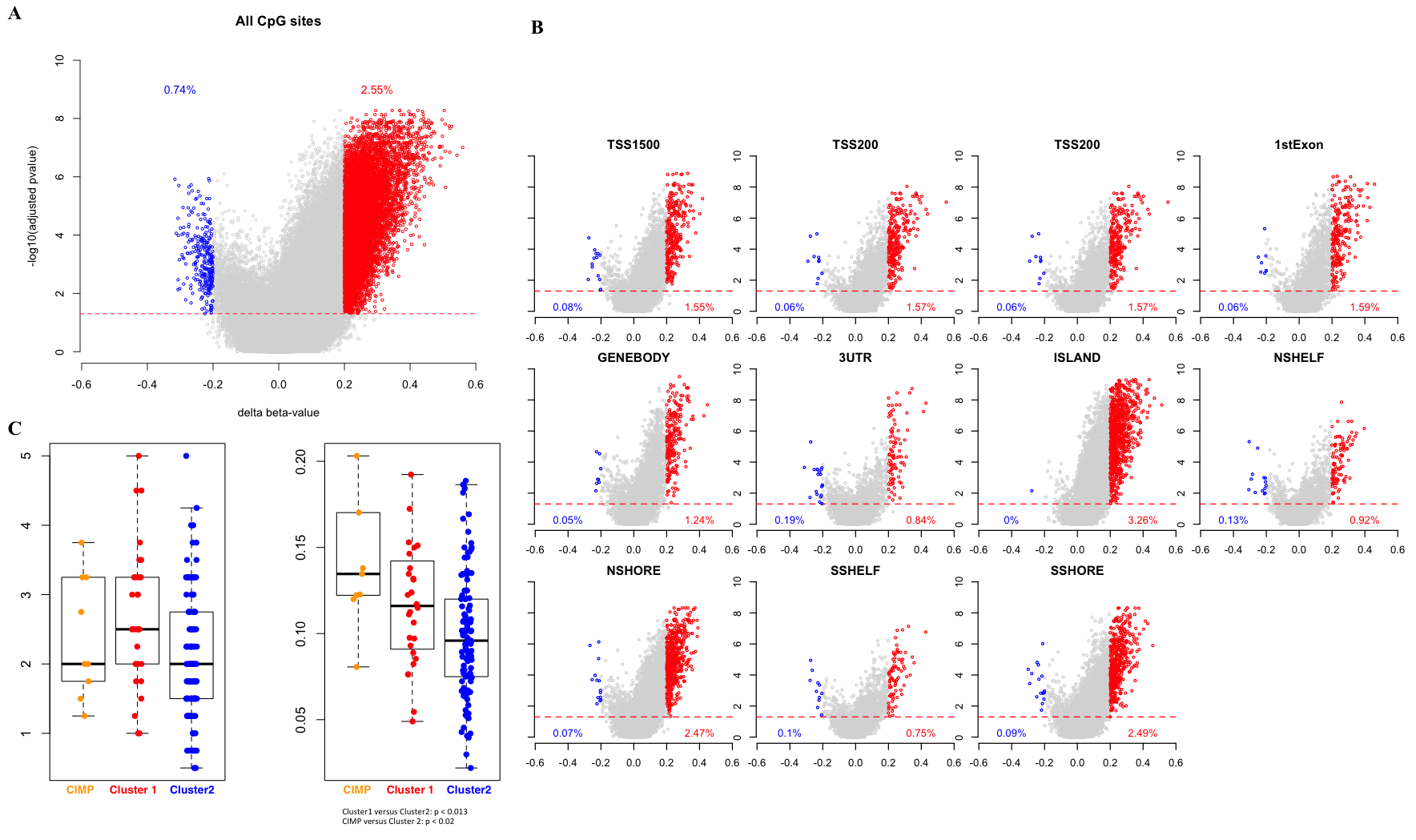

Supplement: S3 Fig — A. Volcano plot of all CpG probe sites between methylation cluster 1 and 2. Differences in mean beta values are shown on x-axis and log transformed p-values (rank sum test) are shown on y-axis. Red dashed line represents 0.05 significance level. B. Volcano plot of CpG probe sites between methylation cluster 1 and 2 after grouped by functional regions. Differences in mean beta values are shown on x-axis and log transformed p-values (rank sum test) are shown on y-axis. Red dashed line represents 0.05 significance level. Annotation details please refer to the R “IMA” package [3]. C. Comparison of C>T in CpGs mutation counts (per millions) and fractions in pRCC WXS set among three different methylation clusters. CIMP: CpG island methylation phenotype. Cluster 1 versus Cluster 2, p < 0.013; CIMP versus Cluster 2: p < 0.02 (rank sum test). (TIF) [file pgen.1006685.s003.tif]

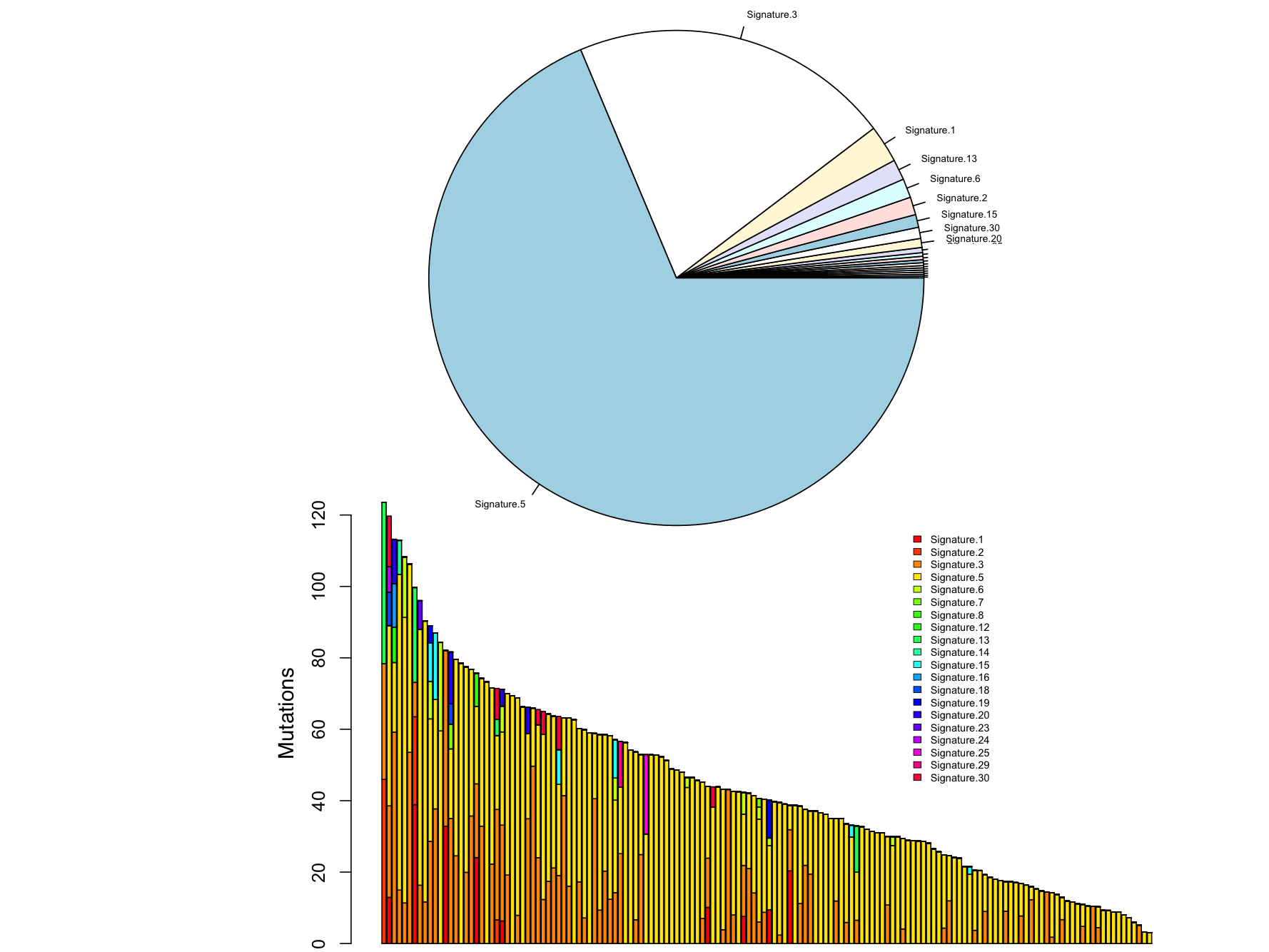

Supplement: S4 Fig — Upper panel: pie chart of signatures contribution percentages by pooling all samples. Signatures contribute less than 5% were not shown. Lower panel: bar plot shows signature distribution in each individual sample. The results grossly agreed with previous results [4] with minor disparity in signature 3. A few samples with no detectable signature are not shown, mostly because they have too few mutations. (TIF) [file pgen.1006685.s004.tif]

**APOBEC3A mRNA expression level**

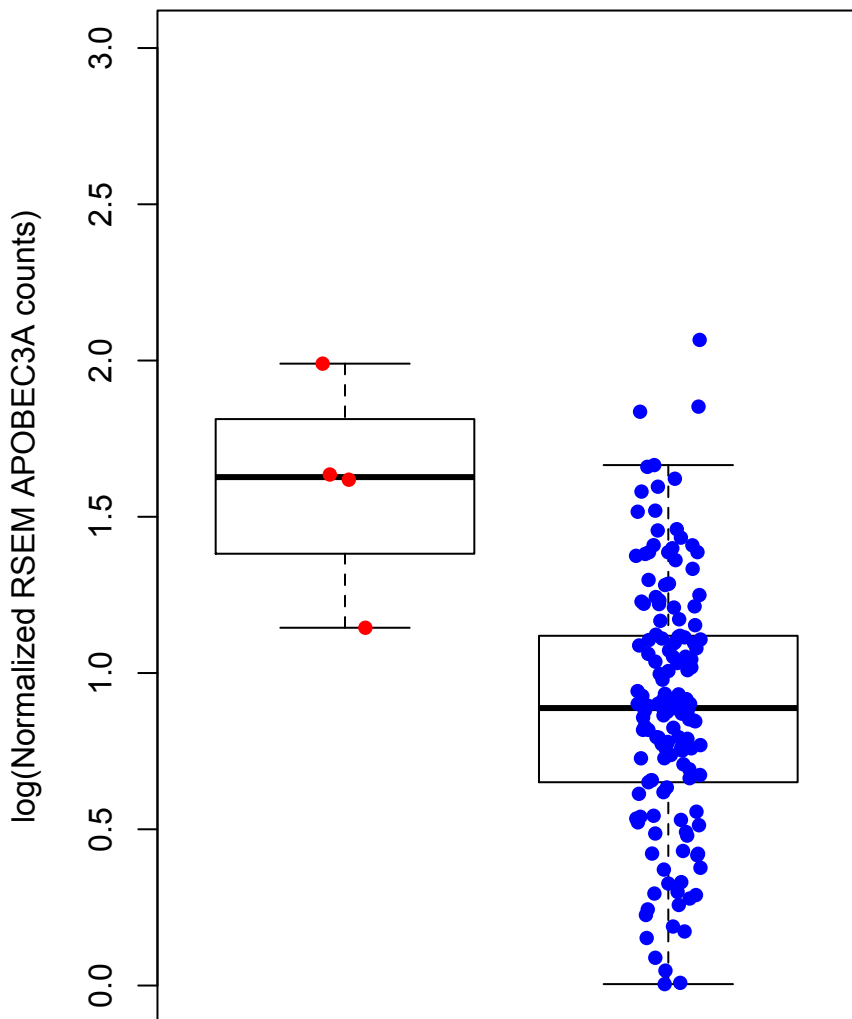

**APOBEC3B mRNA expression level**

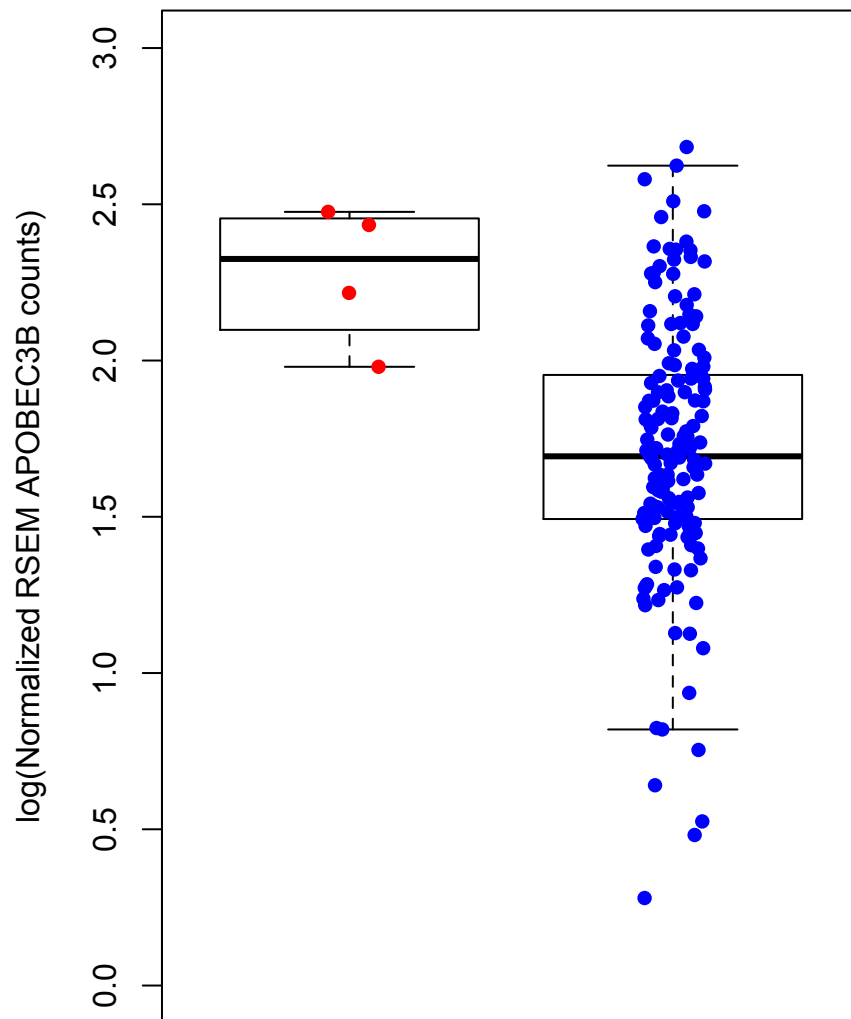

Supplement: S5 Fig — The expression levels of APOBEC3A and APOBEC3B are significantly higher in samples carry APOBEC signatures (red) than the ones do not (blue). p < 0.0022 and p < 0.0039 respectively, one-sided rank sum test. (PDF) [file pgen.1006685.s005.pdf]

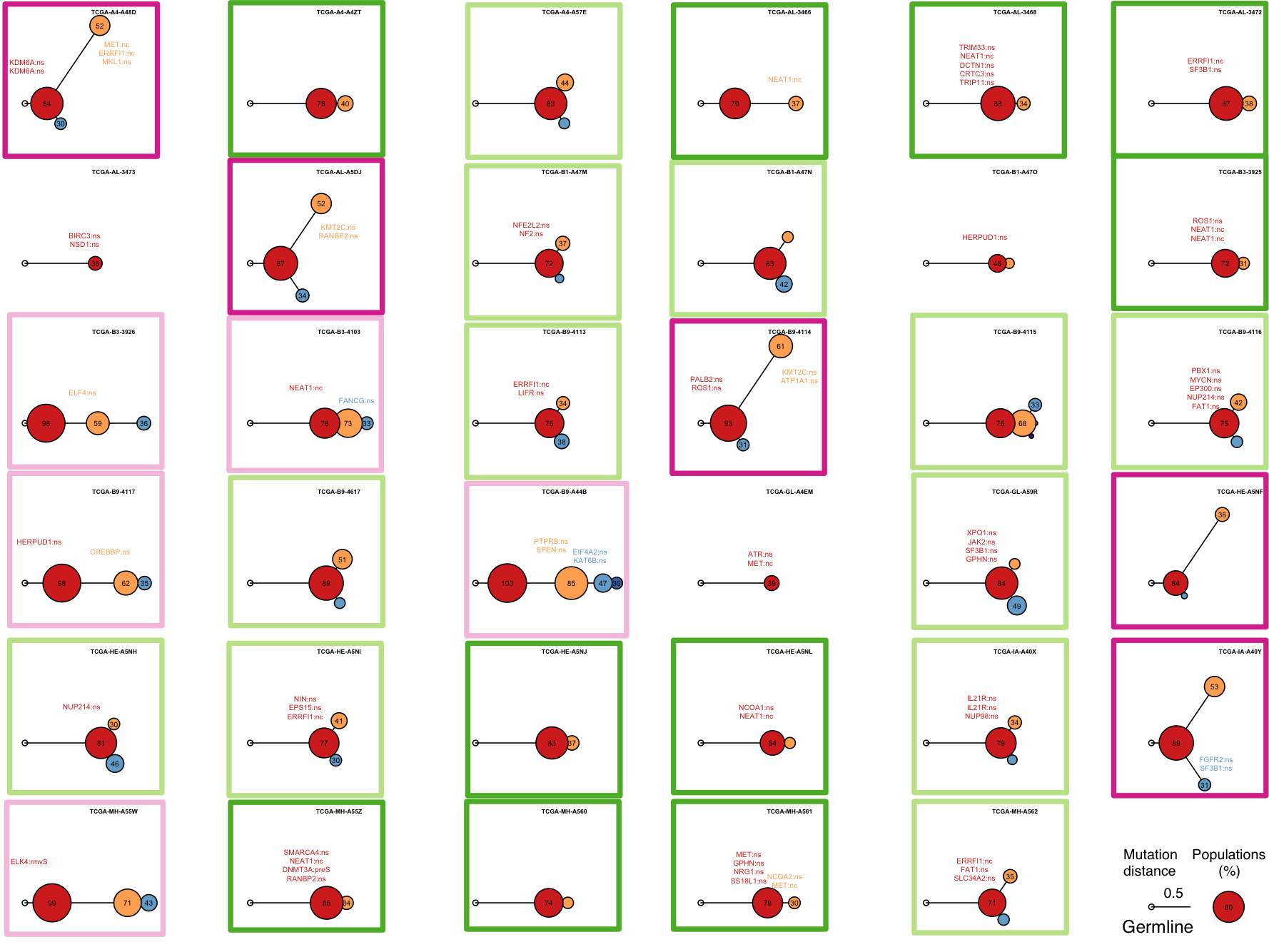

Supplement: S6 Fig — Compared to “wild type” tumors (black), samples with chromatin remodeling gene mutations (orange) had higher percentage of mutations in the early replicating regions. One-sided rank sum test. (TIF) [file pgen.1006685.s006.tif]

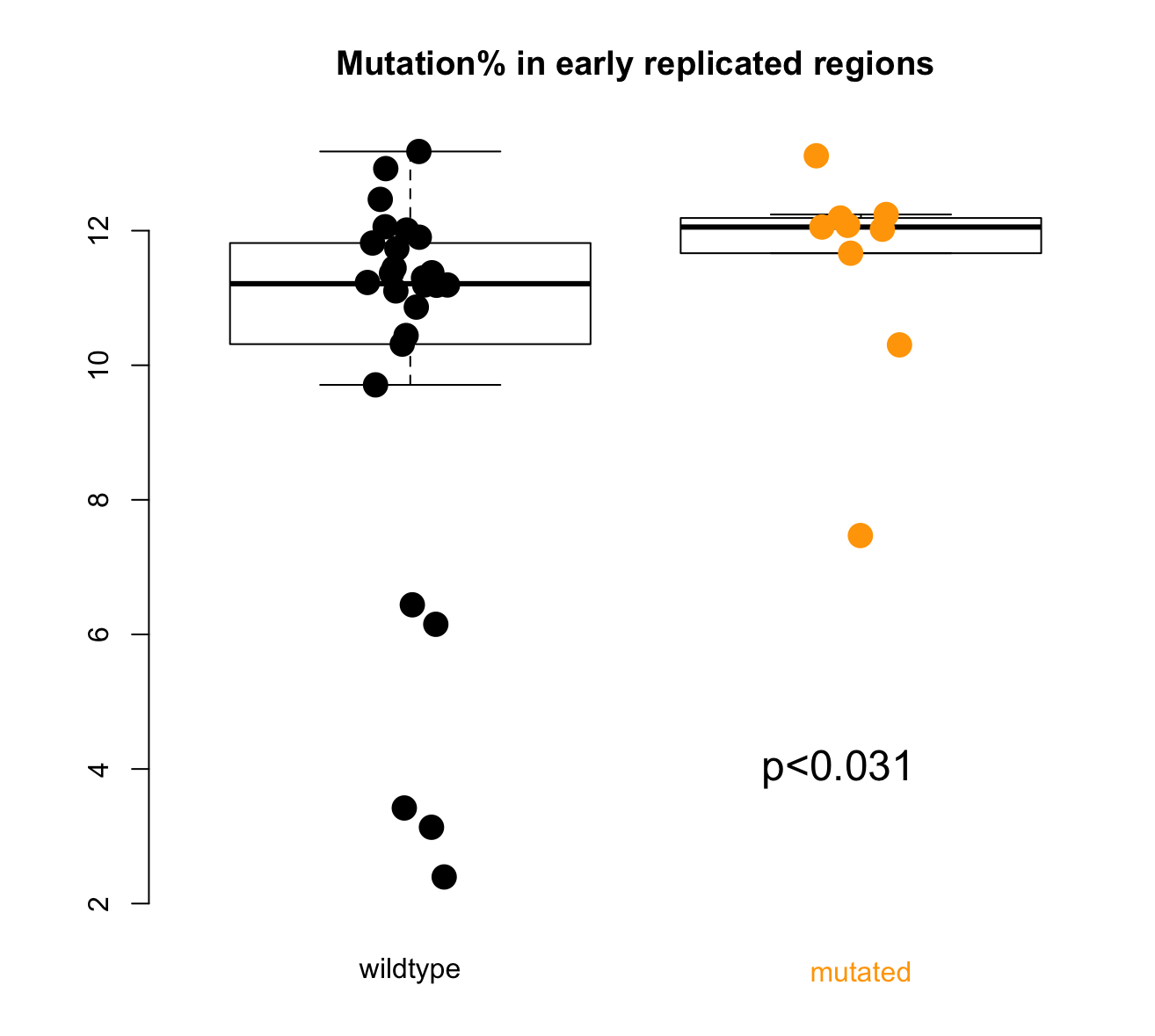

Supplement: S7 Fig — Frame colors indicates four different topology types (See Fig 4). Mutations in cancer-related gene are shown in colors corresponding to where they first appear. Three trees without frame are the ones with a largest population fraction <0.5, indicating unreliable inference of tree structures (due to low mutation counts, sequence error and/or particularly high copy number variation etc.). They were excluded from downstream analysis. (TIF) [file pgen.1006685.s007.tif]
