# Supplementary material for: Whole-genome analysis of papillary kidney cancer finds significant noncoding alterations
Source: PLoS Genet. 2017 Mar 30;13(3):e1006685. doi: 10.1371/journal.pgen.1006685 (PMC5391127; doi:10.1371/journal.pgen.1006685)
Supplement: S1 Reference — (DOCX) [file pgen.1006685.s012.docx]

**Reference**

1. Gao J, Aksoy BA, Dogrusoz U, Dresdner G, Gross B, Sumer SO, et al. Integrative analysis of complex cancer genomics and clinical profiles using the cBioPortal. Science signaling. 2013 Apr 2;6(269):pl1.
2. Cerami E, Gao J, Dogrusoz U, Gross BE, Sumer SO, Aksoy BA, et al. The cBio cancer genomics portal: an open platform for exploring multidimensional cancer genomics data.
3. Wang D, Yan L, Hu Q, Sucheston LE, Higgins MJ, Ambrosone CB, et al. IMA: an R package for high-throughput analysis of Illumina's 450K Infinium methylation data. Bioinformatics. 2012;28(5):729-30.
4. Alexandrov LB, Nik-Zainal S, Wedge DC, Campbell PJ, Stratton MR. Deciphering signatures of mutational processes operative in human cancer. Cell reports. 2013;3(1):246-59.
